# The Role of Community Science in DNA‐Based Biodiversity Monitoring

**DOI:** 10.1111/mec.70100

**Published:** 2025-09-12

**Authors:** Carolina Corrales, Karolina Bacela‐Spychalska, Elena Buzan, Torbjørn Ekrem, Sónia Ferreira, William Goodall‐Copestake, Elaine van Ommen Kloeke, Peter M. Hollingsworth, Sarah J. Bourlat

**Affiliations:** ^1^ Centre for Biodiversity Monitoring and Conservation Research Leibniz Institute for the Analysis of Biodiversity Change, Museum Koenig Bonn Bonn Germany; ^2^ Department of Invertebrate Zoology and Hydrobiology University of Lodz Lodz Poland; ^3^ Faculty of Mathematics, Natural Sciences and Information Technologies University of Primorska Koper Slovenia; ^4^ Faculty of Environmental Protection Velenje Slovenia; ^5^ Department of Natural History, NTNU University Museum Norwegian University of Science and Technology Trondheim Norway; ^6^ CIBIO, Centro de Investigação em Biodiversidade e Recursos Genéticos, InBIO Laboratório Associado Campus de Vairão, Universidade do Porto Vairão Portugal; ^7^ BIOPOLIS Program in Genomics, Biodiversity and Land Planning, CIBIO, Campus de Vairão Vairão Portugal; ^8^ Royal Botanic Garden Edinburgh Edinburgh UK; ^9^ Naturalis Biodiversity Center Leiden the Netherlands

**Keywords:** biodiversity monitoring, community science, DNA‐based methods, hobby experts

## Abstract

The mutual interest in nature by the general public and scientists has led to many collaborations, past and present. Community science shows great potential for monitoring species occurrences and distributions, especially in combination with scalable and (semi)‐automated methods such as DNA‐based monitoring, helping to obtain data from a broader geographic and temporal range than would be possible by the scientific community alone. Here, we present an overview of the complementarity between community science and DNA‐based biomonitoring through examples from ongoing projects. The involvement of hobby experts is particularly crucial for building up the necessary species reference databases that enable DNA‐based monitoring. Based on this overview, we identify some key points related to learning opportunities and participant recognition to maximise the success, impact and benefit of community participants in DNA‐based monitoring.

## 1 Introduction

Biodiversity loss is occurring rapidly due to several anthropogenically driven factors such as climate change, land‐use change, pollution and overexploitation (Pocock et al. 2024; Roy et al. 2024). To address declines in biodiversity, the Convention on Biological Diversity adopted the Kunming–Montreal Global Biodiversity Framework during COP15, aiming to protect and restore biodiversity by 2030 (Roy et al. 2024; Stammnitz et al. 2024). Achieving these goals relies on biomonitoring, an essential tool to track changes in biodiversity (Backstrom et al. 2024), which provides information on species distributions and changes in abundance (Leerhøi et al. 2024) and assesses the efficacy of management interventions and restoration efforts.

However, to have the desired reach and impact, biomonitoring needs to be both massively scaled up by using automated methods as well as carried out not only by scientists, but by a larger cross‐section of society as a shared responsibility. Recently, high‐tech methods have been developed to complement traditional biomonitoring, such as the use of bioacoustics, camera trapping, drones, sensor networks, imaging spectroscopy, satellite data and DNA‐based methods (de Groot et al. 2022; Schweiger and Laliberté 2022; Sheard et al. 2024; Tosa et al. 2021; van Klink et al. 2022; Wägele et al. 2022; Zirngibl et al. 2022). In addition, community science or citizen science (CS) programmes have been developed to enable collaboration networks for carrying out biodiversity surveys (Burian et al. 2023; Chandler et al. 2017; Couton et al. 2023; Johnston et al. 2023; Knudsen et al. 2023; Miya et al. 2022; Moersberger et al. 2024; Pocock et al. 2018; Roy et al. 2024; Suzuki‐Ohno et al. 2023; Zhang et al. 2023). We define CS, for our purpose, as the active participation of non‐professional volunteers (e.g., Indigenous peoples, local communities and general public) in scientific research and biodiversity monitoring by gathering data and conducting experiments under professional guidance (Johnston et al. 2023; Leerhøi et al. 2024; Pernat et al. 2024; Pocock et al. 2024; Roy et al. 2024; Zhang et al. 2023).

CS has gained popularity in environmental and ecological research projects (Fraisl et al. 2022), as it has great potential for monitoring species occurrences and distributions, along with other biodiversity indicators (Chandler et al. 2017; Pereira et al. 2013), and detecting threats (Pocock et al. 2024) over large spatial and temporal scales (Pernat et al. 2024; Suzuki‐Ohno et al. 2023; Tyagi et al. 2023). Moreover, Danielsen et al. (2005) revealed that community monitoring is cheaper than traditional professional monitoring, leading to immediate actions to protect habitats and manage resources more sustainably. Even the Global Biodiversity Framework suggested engaging community scientists in biomonitoring plans to implement and accomplish their goals (Danielsen et al. 2024).

CS participants can have a contributory or a collaborative role (Haklay 2013; Pocock et al. 2018; Senabre Hidalgo et al. 2021). The former refers to sample/data collectors, and the latter involves co‐creation CS approaches, which provide a two‐way interaction between researchers and CS participants by allowing the latter to be involved in the project design and implementation (Bela et al. 2016; Kelly et al. 2020) (for examples, see Tøttrup et al. 2025). In this manner, CS will transition from an extractive to a reciprocal relationship (Hall et al. 2024). So far, CS contributions to biomonitoring have been mainly restricted to visual observations and the collection of samples and their associated data (Forsblom et al. 2024; Hall et al. 2024; Roy et al. 2024) (Table S1).

Following best practices (Bonney et al. 2009; Chase and Levine 2016; ECSA 2015), CS participation could be extended to (1) volunteers suggesting improvements and proposing questions of most interest to them to contribute to project design and (2) complementing and enriching the role of scientists by assisting in every step of the scientific process, leading to results that benefit the community (Clarke et al. 2023; Danielsen et al. 2024; Gardiner and Roy 2022; Hall et al. 2024; Leerhøi et al. 2024; Roy et al. 2024; Senabre Hidalgo et al. 2021; Schmeller et al. 2017). The latter is critical when Indigenous peoples and local communities have been involved (McCartney et al. 2023; Tengö et al. 2021), as their knowledge about the ecological systems is valuable and should not be neglected (Hall et al. 2024). Specifically in projects leading to the design of conservation programmes, implementation may be expected to have greater success if they are better understood by the community because they were involved in the process that resulted in the development of a specific regulation, as well as in the monitoring of the conservation action results (McKinley et al. 2017). Furthermore, the more engaged volunteers are in the process, the more likely they are to benefit from any training, leading to more robust data, which in turn will reduce the need for validation and increase satisfaction and motivation to participate.

## 2 DNA‐Based Methods for Biomonitoring

There are several DNA‐based methods (e.g., environmental DNA methylation, shotgun metagenomics, environmental RNA metatranscriptomics and CRISPR‐based methods) that could increase the scope of biomonitoring (Ahi and Schenekar 2025; Balard et al. 2024; Curto et al. 2024; Durán‐Vinet et al. 2024, 2025; Hempel 2022; Yates et al. 2021). However, metabarcoding is the most commonly used high‐throughput sequencing technology for environmental DNA (eDNA) and bulk sample analyses (Lacoursière‐Roussel and Deiner 2021; Martins et al. 2019; Meyer et al. 2021; Yu et al. 2012), followed by species‐specific detection methods (e.g., quantitative PCR) (Biggs et al. 2015; Feng and Lougheed 2023; Melliti et al. 2025; Osathanunkul 2024). eDNA is the mix of both intra‐ and extracellular DNA from microbial and macrobial organisms present in an environmental sample such as water, sediment, soil, or air, as well as plant and other surfaces (Lacoursière‐Roussel and Deiner 2021; Pawlowski et al. 2020). Bulk samples are mass‐collected specimens or community samples (e.g., Malaise traps) used to extract DNA from these organismal pools. These samples have different collection techniques and spatial scales (i.e., bulk samples are collected locally, whereas eDNA can disperse over large distances) that can capture different invertebrate community compositions; hence, it is recommended to use them in parallel (Doloiras‐Laraño et al. 2023; Gleason et al. 2021; Macher et al. 2018; Múrria et al. 2024).

DNA‐based methods are reliable and very well established (Handley et al. 2023), yet they are not regularly used by environmental managers and policymakers to monitor populations and species trends nor for conservation actions (Laamanen et al. 2025; Moersberger et al. 2024), except for some European, North American and Australian agencies (e.g., UK Environmental Observation Framework, Fisheries and Oceans Canada, The US Fish and Wildlife Service, National Oceanic and Atmospheric Administration and the Western Australian Biodiversity Science Institute) that do apply this methodology on an ongoing basis (Altermatt et al. 2025).

It is known that DNA‐based methods provide several advantages including (1) identification of understudied, cryptic and difficult‐to‐observe species that cannot be detected through conventional monitoring (Cunningham‐Eurich et al. 2023; Knudsen et al. 2023; Krehenwinkel et al. 2024; Odah 2024; Zhang et al. 2023), (2) detection of non‐indigenous or invasive species (Holman et al. 2019; Larson et al. 2020), (3) taxonomic assignment through reverse taxonomy when morphological identification is difficult or specimens are lacking (Pereira‐da‐Conceicoa et al. 2021), as long as well‐curated reference libraries are available, which are almost complete for well‐known taxa from the Global North, but still incomplete in biodiversity hotspots or for poorly studied taxa (e.g., nematodes) (see Weigand et al. 2019) and (4) potential to increase the scale, speed and scope of biodiversity monitoring as they tend to deliver fewer errors and reduce processing time compared to morphological identifications, which are user dependent (Pereira‐da‐Conceicoa et al. 2021).

Integrating both DNA‐based methods with CS can help address four major obstacles that traditional biodiversity monitoring methods face: (1) the use of invasive methods which are expensive and labour‐intensive (Burian et al. 2023; Knudsen et al. 2023; Lynggaard et al. 2019; Zhang et al. 2023); (2) targeting a handful of species compared to total biodiversity, which is often biased towards iconic taxa (Baillie et al. 2008; Hill et al. 2021; Moussy et al. 2022; Schmeller et al. 2017); (3) poor spatial and temporal coverage (Moersberger et al. 2024; Pernat et al. 2024; Roy et al. 2024) and (4) taxonomic gaps (Henter et al. 2016; Macher et al. 2018; McKinley et al. 2017; Odah 2024).

Especially for aquatic ecosystems (Altermatt et al. 2025; DiBattista et al. 2021; Kelly‐Quinn et al. 2023; Suter et al. 2023; Zhang et al. 2023), CS has helped professional scientists to cover large sampling/monitoring areas and provide long‐term data (Backstrom et al. 2024; Cunningham‐Eurich et al. 2023; Knudsen et al. 2023; Suter et al. 2023) or to sample several locations simultaneously to reduce the effect of temporal variations (Agersnap et al. 2022; Stammnitz et al. 2024). Yet, very few studies have combined DNA‐based methods with CS approaches (Agersnap et al. 2022; DiBattista 2023; Kelly‐Quinn et al. 2023; Knudsen et al. 2023; Leerhøi et al. 2024).

## 3 Engaging Community Scientists in DNA‐Based Biomonitoring

Nowadays, it has become easier and quicker for the general public to opportunistically contribute to biomonitoring due to the rise of online interactive tools and biodiversity platforms to capture species occurrences in the field in real‐time (e.g., iNaturalist, observation.org, eBird and Mushroom Observer) (Arazy and Malkinson 2021; Della Rocca et al. 2024; Lemmens et al. 2021; Sheard et al. 2024; Speelman et al. 2023; Uche‐Dike et al. 2024; Van Vliet and Moore 2016). Such easy‐to‐use tools have lowered the participation threshold in CS projects and further increased the potential scale and robustness of biodiversity datasets (e.g., GBIF). However, the general public is not yet fully aware of the potential of eDNA in biodiversity monitoring (Barbaccia et al. 2025; Clarke et al. 2023).

Agersnap et al. (2022), Biggs et al. (2015), Clarke et al. (2023), Meyer et al. (2021) and Tøttrup et al. (2021), among others, demonstrated that community scientists can provide high‐quality samples for eDNA analyses as long as researchers provide ongoing learning and collaboration opportunities (Bela et al. 2016; Gardiner and Roy 2022). CS projects (Table S1) usually provide educational and interactive training in the form of workshops, webinars, or videos designed to explain the aims of the projects, the sampling and storage instructions, collection of metadata, use of sampling kits and equipment and the difficulties associated with sampling (e.g., air bubble clogging filters) along with the risks they may endure (e.g., bad weather conditions and venomous animals) (Broadhurst et al. 2023; Burian et al. 2023; Clarke et al. 2023; Knudsen et al. 2023; Kvalheim et al. 2024; Meyer et al. 2021; Miya et al. 2022; Moersberger et al. 2024; Shan et al. 2023; Suzuki‐Ohno et al. 2023; Tøttrup et al. 2021). Face‐to‐face training in the field and hands‐on activities are also crucial to enhance understanding of the tasks undertaken and facilitate mutual exchange of knowledge and scientific insights (Bela et al. 2016; Clarke et al. 2023; Kasten et al. 2021; Knudsen et al. 2023; Peter et al. 2021; Zhang et al. 2023). Follow‐up meetings and guidelines can clarify expectations to help maintain participation and trust throughout projects. Social interaction among participants via social media, email/phone consultations, wikis, or further workshops can provide additional opportunities for information exchange and technical assistance (Agersnap et al. 2022; Burian et al. 2023; Sheard et al. 2024).

CS initiatives involve several logistical considerations that should be addressed during the planning process; hence, many recommendations are in place (Bonney 2021; García et al. 2021; Goudeseune et al. 2020; Pateman et al. 2025). For instance, researchers should determine which group they aim to engage (e.g., stakeholders or the general public). Additionally, it is essential to define the geographic scope and sampling locations to avoid biased geographic sampling, establish the sample type/number of replicates, sampling methods and sampling kits to be used and the metadata to be collected (e.g., collection date, collector's name, location, geographical coordinates and sampling method) (Agersnap et al. 2022; Clarke et al. 2023; Leerhøi et al. 2024; Shan et al. 2023). Note that choosing the right preservation method depends on project logistics (e.g., transport procedures) and the methods used for downstream extraction (Agersnap et al. 2022; Meyer et al. 2021; Rodriguez et al. 2025; Seymour et al. 2024). Furthermore, it is important to assess the need for sample collection permits for the participants, the suitability of current databases, and to decide how results will be shared with the public (Meyer et al. 2021; Seymour et al. 2024).

### 3.1 Games as a Learning Tool

Games have been designed for training and education (Sandbrook et al. 2015), and they can reinforce the understanding, motivation and active involvement of participants in CS approaches that introduce new methods (Sajan and Sapkota 2024), fostering a learning environment (Othman and Ching 2024). For example, the Biodiversity Genomics Europe (BGE) project has developed and adapted various games (e.g., ‘DNA detectives’ from Kerr and Breitbart (2021)) to explain the basic concepts of DNA barcoding and the use of genomic tools to monitor biodiversity. Other games available to the public include BarcodingGO, which teaches eDNA and bioinformatic concepts (de Nunes et al. 2021) and the Brickopore, which explains the fundamental concepts and principles of DNA sequencing using LEGO bricks (Boulter et al. 2022). Participating in these games allows CS participants to come into contact with concepts and gain practice articulating them through the scientific method and the projects in development. Participants also gain valuable insights into the methodologies used and take part in creating hypotheses and identifying expected outcomes.

## 4 Going Beyond Sampling: Learning Opportunities and Co‐Creation

DNA‐based sampling methods are straightforward to follow (Altermatt et al. 2025; Bohmann et al. 2014), especially when kits, eDNA CS samplers (e.g., Smith‐Root) or state‐of‐the‐art drones (Aucone et al. 2023; Geckeler et al. 2025; Kirchgeorg et al. 2024) are provided. CS participants, however, are often unfamiliar with subsequent tasks, such as lab work and data analyses (Erasmus 2021; Florio et al. 2018; Henter et al. 2016; Steinke et al. 2017; Tosh et al. 2016). Activities extending beyond the collection of samples (Table 1), along with adequate training, are desirable to enhance the volunteers' motivation and learning in molecular biology and to contribute to high‐value biodiversity data (Clarke et al. 2023; Kasten et al. 2021; Leerhøi et al. 2024; Wright et al. 2024 ).

**TABLE 1:** Examples of potential actions undertaken by professional scientists to further engage community scientists beyond sampling.

Action	Considerations
Mentorship	Project leaders or PhD students can be role models for CS participants. Consider organising virtual or in‐person meetings, and engaging in social media and learning management platforms for educational support and guidance, according to the participants' needs and expectations (Chiovitti et al. 2019; Cho et al. 2021; Polidoro and Clement 2018)
Face‐to‐face fieldwork	Consider producing a short instructional video or introductory reading material to get the necessary background before going to the field (Agersnap et al. 2022; Clarke et al. 2023) Consider producing illustrated guidelines or protocols showing step‐by‐step procedures to avoid misinterpretations and standardised instructions that can be reused across projects. Translating into distinct languages will allow wider use. Be careful using technical terms. These can be replaced by more common terms, or a glossary can be included (Clarke et al. 2023; Izaguirre 2024) Consider organising a bioblitz‐like event to reinforce field methods, involving hobby experts or experienced para‐taxonomists or design a ‘train the trainers’ course if necessary (Biggs et al. 2015; Hupało et al. 2021; Meyer et al. 2019, 2021) Consider partnering with eDNA service providers (e.g., NatureMetrics, SpyGen, Wilderlab, VigiDNA network and SimplexDNA) that already have experience with community eDNA projects, offer sampling strategy designs and sampling training and provide standardised sampling kits (Ayre et al. 2025; Clarke et al. 2023; Roberts et al. 2025)
Training in lab work and data analysis	Implement workshops/courses that can provide new skills as well as inspiration to get involved in science (Leerhøi et al. 2024). These can be taught by the project leader, PhD students or educators with molecular biology training (Polidoro and Clement 2018) Take advantage of online resources (e.g., DNA barcoding 101, DNA learning centre, DNA barcoding‐Australian Museum, GBIF biodiversity data, EMBL, iBOL and EPA New Zealand) to present new concepts ahead and ensure the same knowledge level, so the focus can be on hands‐on exercises. Some of these centres also offer in‐person training Consider collaborating with teaching and learning programmes or community science labs to develop your activities (e.g., Wellcome Connecting Science, EMBL science education, DNA learning centre, Gene Technology Access Centre, eDNA Collaborative, Genspace, BioCurious and BioBus) (Seymour et al. 2024) Provide demonstrations showcasing the features of friendly tools, such as the CALeDNA‐DNA Explorer, to analyse data (Miyatsu 2023)
Games	Consider the use of games to explain scientific concepts, address scientific problems and develop scientific thinking (de Nunes et al. 2021) Produce games with specific goals that are simple, clear and fun, to avoid losing the participant's motivation Consider the preferences and skills of players: Distinct games for distinct levels and publics

Some institutions, such as the Cold Spring Harbour Laboratory (DNA Learning Centre), natural history museums (NHM) and universities offer CS participants first‐hand experience of laboratory work, supporting science literacy (Dopico et al. 2021; Leerhøi et al. 2024; Meyer et al. 2021). For example, Chiovitti et al. (2019) and Wright et al. (2024) trained school teachers in DNA barcoding, Seymour et al. (2024) conducted sessions on standard amplicon library preparation and gel electrophoresis methods for PhD candidates and stakeholders, while Tøttrup et al. (2025) trained high school students on molecular methods for analysing archaeological materials. Before bringing participants into a molecular lab, it is essential to ensure proper supervision, provide biosafety training and appropriate personal protective equipment and to use non‐toxic products if possible (Dopico et al. 2021). Mobile sequencing labs, including portable labs (e.g., BenthoLab and Biomeme) and portable sequencing devices (e.g., MinION) are playing an increasingly important role, as they not only make advanced technology accessible to everyone but also boost the involvement of CS participants in remote genetic surveys by allowing the analyses and self‐assessment of their own samples (Stammnitz et al. 2024). Moreover, it is now possible to send samples to commercial laboratories for analysis thanks to falling costs (Clarke et al. 2023). For participants who are interested in understanding all stages of a DNA‐based project, a more extensive training could be offered in the form of lectures, including bioinformatics, taxonomic assignment and the use of databases as suggested by Seymour et al. (2024).

One example of a successful co‐creation approach is the ‘Original DNA & life’ and the ‘Extreme DNA & life’ projects, based at the NHM of Denmark and the University of Copenhagen, and in collaboration with the Danish National Union of Upper Secondary School Teachers. These pioneering projects that aim to monitor aquatic environments have involved more than 3000 high school students, who collected water samples, prepared eDNA analyses using qPCR methods and conducted data analyses together with teachers and researchers (Leerhøi et al. 2024; Tøttrup et al. 2021 ). Biology teachers are offered a course on the use of lab equipment and methodologies. Under their guidance, students can conduct lab work independently in one of the local laboratories established by the project at two different high schools. A website was also set up, including all protocols necessary for DNA analysis (https://snm.dk/da/artikel/dna‐paa‐forkant, in Danish). This kind of project proves that public engagement and awareness of biodiversity can be enhanced if eDNA studies are integrated into educational programs (Barbaccia et al. 2025; Meyer et al. 2021).

Co‐creation requires established partnerships with different entities/stakeholders, such as environmental agencies, natural history museums, universities, schools, environmental NGOs, conservation charities, hunting associations, fishing clubs, local/national scientific societies and farmers, among others, to promote CS activities and recruit participants, as well as to provide long‐term sustainability (Agersnap et al. 2022; Clarke et al. 2023; Leerhøi et al. 2024; Meyer et al. 2021; Tøttrup et al. 2025). Community labs or do‐it‐yourself biology communities can also be considered for CS approaches as they already have a scientific background and can provide physical laboratories, resources and education (Aldulijan et al. 2025; Eireiner 2025; Landrain et al. 2013). Guasch et al. (2022) and Clarke et al. (2023) suggested some recommendations for co‐creation projects, such as understanding and combining local expertise and knowledge, ensuring a wide range of perspectives, obtaining feedback from the CS participants, designing well‐structured work sessions and providing different multi‐sensory materials (e.g., pictures and videos) to reduce unnecessary mental effort. In addition, Stevens et al. (2014) and Hoyte (2021) provide lessons for researchers and practitioners looking to engage in co‐creation approaches with Indigenous communities.

## 5 CS in the Global‐South

Wealthy countries, such as New Zealand, Australia, the United Kingdom, Denmark and the United States, are leading in the development of innovative CS programmes using eDNA. eDNA studies in the Global South are currently limited, with community scientists mostly contributing occurrence records, while researchers perform eDNA procedures (Belle et al. 2019; Cortelezzi and Paz 2023; Cruz‐Cano et al. 2024; Huerlimann et al. 2020; Quilumbaquin et al. 2023; Stephenson et al. 2022; von der Heyden 2023). The application of eDNA in this region faces several challenges, including a lack of funding and expertise, a lack of taxonomic/geographic resolution in databases and inadequate data‐sharing practices (Altermatt et al. 2025; Capurso and Stewart 2024; Gonzalez 2023; Stephenson et al. 2022; von der Heyden 2023). The Global North can collaborate and support the Global South by building long‐term local capacity and enhancing laboratories and infrastructure (Lopes‐Lima et al. 2025; Sahu et al. 2025; von der Heyden 2023). For instance, a collaboration between France and Colombia led to the installation of the first mobile eDNA lab at the Colombian Institute of Marine and Coastal Research (INVEMAR) in 2022, including also training of INVEMAR scientists by SPYGEN teams to carry out eDNA analysis (https://www.vigilife.org/en/mobile_laboratory/, accessed on 5 May 2025). Schmeller et al. (2017) defined a framework for capacity building in biodiversity monitoring that can be put into practice in the Global South.

Another promising CS approach that can contribute to closing biodiversity gaps in the Global South is nature tourism (Martin et al. 2025). Under the guidance of local experts, tourists can have the opportunity to take part in scientific research while simultaneously learning about the biodiversity of the visited locations (Paschoalini Frias et al. 2025). Besides, integrating opportunistic eDNA sampling into tours provides high spatiotemporal resolution and long‐term data of monitored species (Rodriguez et al. 2025). Several tourism operators have partnered with eDNA service providers, as well as organisations like IUCN and WWF, to engage tourists in biodiversity monitoring through eDNA sampling across the world. For instance, tourism operators in Peru were involved in the collection of 32 water samples in different Amazon rivers, identifying 250 vertebrate species (https://www.wwf.org.pe/?361095/Citizen‐Science‐sustainable‐tourism‐contributes‐to‐the‐conservation‐of‐river‐dolphins‐and‐other‐aquatic‐species‐in‐Northern‐Amazon, accessed on 5 May 2025). A North American travel agency has provided 78 global samples, which are used to help build the IUCN eBioAtlas database (https://www.exodustravels.com/insights/venture‐out‐on‐our‐citizen‐science‐departures, accessed on 13 Aug. 2025), while whale‐watching tours in the Azores Islands, Iceland and Italy are involved in the collection of water samples for cetacean monitoring (Barbaccia et al. 2025; Rodriguez et al. 2025).

Community groups (e.g., Macrolatinos), local stakeholders, NGOs (e.g., WWF, Earthwatch and WCS) and consortia (e.g., iBOL and CS Network for the Amazon) can help in the integration of CS into DNA‐based methods by adapting this tool to local needs, developing sampling kits and standardising sampling protocols and providing long‐term funding (Sahu et al. 2025). This, in turn, might become a solution for completing thorough sampling campaigns in time and space, for collecting specimens to fill gaps in DNA reference libraries and for self‐sustainable biomonitoring of remote and inaccessible areas (Huerlimann et al. 2020), empowering local communities to monitor their own biodiversity (Stephenson et al. 2022) (Table S2).

## 6 A Special Group of Community Scientists: Hobby Experts and Their Role in Closing Taxonomic Gaps

DNA‐based monitoring approaches, especially eDNA, can help fill gaps in species inventories and reduce the reliance on taxonomists (Bohmann et al. 2014; Cortelezzi and Paz 2023). However, these molecular techniques rely on accurate and well‐curated DNA barcode reference libraries for species validation, which are developed and improved by taxonomists (Altermatt et al. 2025; Janzen and Hallwachs 2011; Laamanen et al. 2025; Sahu et al. 2025). eDNA has been able to detect new species or cryptic species that have not yet been described because they are morphologically similar to already described species, particularly in biodiversity‐rich regions (Mariac et al. 2022; Mena et al. 2021), but misidentifications and deficient taxonomic assignment of OTUs still occur due to poorly curated or incomplete databases (Blackman et al. 2024). Therefore, taxonomists are essential to review and assess the species list produced by eDNA as well as the species occurrence (Blackman et al. 2024; Mena et al. 2021). Without sufficient taxonomic knowledge, the characterisation of biodiversity is precarious, especially when taxonomic assignment at the species level is needed (Apothéloz‐Perret‐Gentil 2017; Schmeller et al. 2017).

In times of taxonomy expertise decline, hobby experts are indispensable for building the knowledge base around species and biodiversity data. Their often vast knowledge and skills in identifying and recording species occurrences, especially when dealing with local and particular taxonomic groups (Peter et al. 2021; Santaoja 2021; Smith 2024), can exceed that available in the professional sector. They alone are responsible for more than 60% of species discoveries worldwide (Fontaine et al. 2012). Moreover, hobby experts significantly contribute to scientific understanding not only by donating literature and specimen collections to NHMs (Sforzi et al. 2018) but also by providing details of natural history and biographical data (Fontaine et al. 2021; Smith 2024).

CS initiatives led by NHMs and local/national scientific societies could help foster taxonomy, acting as a bridge to provide a common scientific path by (1) training younger naturalists to fill gaps in taxonomy research, and in some cases start a professional path (Peter et al. 2021; Santaoja 2021); (2) training the local community to become para‐taxonomists to support biodiversity inventories in their territories (Janzen 2004; Kazmier 2017) and (3) connecting hobby experts with academic taxonomists for collaborations and training in state‐of‐the‐art molecular technologies and novel policies (Fontaine et al. 2021; Lücking 2020; Schmeller et al. 2017; Pearson et al. 2011). Greater involvement of both academic taxonomists and hobby experts in CS initiatives and digital platforms will promote taxonomic knowledge among community scientists, improving their observational and identification skills (Lücking 2020; Mesaglio and Callaghan 2021; Viola et al. 2022). Some projects carried out by NHMs have already harnessed the taxonomic skills of hobby experts through CS initiatives (e.g., GBOL I & II, ARISE and BGE), whereas others focus on building taxonomic research capacity (e.g., Barcoding the Broads, SPRING, EDIT and TETTRIs) (Table S3).

Mentorship within CS initiatives is another alternative that allows transfer of knowledge between younger naturalists/para‐taxonomists and experienced taxonomists (European Commission: Joint Research Centre et al. 2024; Schmeller et al. 2017). During mentorships, mentors can (1) provide or improve the faunistic and molecular skills along with critical thinking, communication and literacy skills of the mentees, (2) support and guide mentees through the scientific learning process and (3) increase mentees' motivation (Hargitai et al. 2022; Minocha et al. 2024).

## 7 Challenges

Although CS approaches highly benefit biomonitoring, there are still several challenges that may hinder CS project accomplishments:

1. **Sample and data reliability**. There is still concern regarding CS sampling precision and data quality due to the different skills and knowledge of the participants (Kasten et al. 2021), which can cause poor data reliability (e.g., spatial bias due to inadequate sampling design, presence‐only data, species bias, false positive errors within species observations and misidentifications) (Callaghan et al. 2023; Crall et al. 2011; Foster‐Smith and Evans 2003; Gorleri et al. 2023; Johnston et al. 2023; Pocock et al. 2024; Probert et al. 2022). Tøttrup et al. (2021) suggested that there is an inherent risk of false‐positive and false‐negative detections, regardless of whether trained staff or a CS participant is performing the eDNA analysis. Polidoro and Clement (2018) suggest that the long‐term conservation benefits of CS exceed these potential drawbacks, which can be reduced by having access to in‐person training and mentoring, and by providing clear, detailed and standardised protocols matching the project's goals along with data collection equipment (Balázs et al. 2021; Gardiner and Roy 2022).
2. **Human resources and time**. CS projects require both time and experience. Professional scientists would need to dedicate a large amount of time to participate in such initiatives and offer comprehensive guidance, mentoring and training to CS participants (Aldulijan et al. 2025; Schmeller et al. 2017). In return, they will benefit by getting more data, increasing public knowledge of their research and contributing to public education (Tøttrup et al. 2021). Building community partnerships is also time‐consuming, but it will lead to opening new collaborations and enabling new research questions (Hallett et al. 2017). Besides, the importance of human interaction in learning and sharing scientific insights cannot be overstated (Seymour et al. 2024).
3. **Funding**. Financial resources are crucial for carrying out CS projects. CS projects can be sponsored by government agencies, national societies and trusts, enterprise/industrial foundations, private foundations, philanthropic organisations, or Open Science national programmes. Usually, project funding is short‐ to medium‐term and mainly granted to researchers, plus projects focused on CS need to find an appropriate evaluator who understands the concept and use of CS in research (Achenbach 2022; Pateman et al. 2025). However, funding bodies, such as the European Commission, are now committed to funding scientific projects that ‘promote co‐creation, co‐design, and co‐assessment (assisting in monitoring of a project) through the engagement of stakeholders and citizen scientists’ (Arentoft 2023), especially of young people, hard‐to‐reach groups and vulnerable population groups. Exclusive CS funding initiatives have also been launched, such as the UK Research and Innovation Citizen Science Exploration Grant, or the Accelarator Impetus that provided funding, training and mentoring to researchers to help them reach the full potential of the project. The Horizon Europe programme recently opened the calls ‘Strengthening the capacity of citizen science in biodiversity observation’ and the Marie Sklodowska Curie Actions Citizen for Science to facilitate direct dialogue between science and society and promote community engagement. Additionally, crowdfunding platforms (e.g., ScienceBooster and Experiment.com) are a non‐traditional funding option that allow project leaders to receive funding from the general public (Sauermann et al. 2019; Wilson 2019). The webinar ‘The Citizen Science Funding Landscape’, organised by the TIME4CS consortium, provides further information on the topic (accessed on 8 July 2025).
4. **Protocols' standardisation**. Sampling protocols should contain detailed and clear step‐by‐step instructions, as CS participants do not usually have previous experience with eDNA methods (Clarke et al. 2023). Rigorous field hygiene practices, plus negative controls to minimise contamination, also need to be addressed (Seymour et al. 2024). Usually, protocols are standardised according to local or regional needs. A good example is the UNESCO standardised DNA sampling booklet that has been used at 21 World Heritage marine sites across 19 countries (Suominen et al. 2024). This protocol includes information and photos regarding prior preparations to the collecting process, sampling kits, metadata collection and sampling and preservation of water samples. Ideally, specific platforms should be used to collect metadata, such as PlutoFGo (Abarenkov et al. 2010), EpiCollect (Aanensen et al. 2009) or KoBoToolbox (Meyer et al. 2021), which greatly simplify data collection in the field by allowing samples, metadata and photographs to be registered in a standardised manner.
5. **Communication**. DNA‐based monitoring methods are complex, and data interpretation requires thorough and clear explanations (Laamanen et al. 2025). Therefore, communication is essential for successful collaborations among parties (i.e., scientists, policymakers, the public and stakeholders) to maintain confidence and trust in the results, manage expectations and make proper management decisions (Hecker et al. 2018; Probert et al. 2022; Toomey 2023), particularly when communicating insights derived from DNA‐based survey approaches (see Stein et al. 2024). Projects, protocols and results should always be presented in the native language, as not everyone is fluent in English. Ideally, complex scientific language should be avoided (Brandt et al. 2022; Frigerio et al. 2021), and both scientific and vernacular names should be used when referring to species (Santaoja 2021). In this way, non‐scientists can better understand their surrounding biodiversity and establish a connection with nature (Altermatt et al. 2025; Suzuki‐Ohno et al. 2023). It is important to note that eDNA data that originated from indigenous or local communities should adopt CARE principles, and that these communities have the right to keep their data private unless they permit it to be made publicly available (Altermatt et al. 2025).
6. **Sustainability**. Collaborative partnerships with monitoring government agencies or industry, developing science hubs in communal spaces and engagement with other projects or communities are essential to create a sustainable CS approach (Leerhøi et al. 2024; Sauermann et al. 2020). Biodiversity Observatory Networks can also provide support and link to other biomonitoring projects.
7. **Learning outcomes**. So far, investigations regarding the learning outcomes of the participants are lacking (Brandt et al. 2022; Tøttrup et al. 2021). Yet, surveys, such as retrospective questionnaires, can offer valuable insights into the success of conservation messages and help identify pitfalls in citizen science initiatives by evaluating how the participants' knowledge and behaviour change after taking part in a CS activity (Barbaccia et al. 2025; Bonicalza et al. 2024).

## 8 Recognition

Motivational factors such as curiosity, desire to gain new skills and understand the natural world, or willingness to contribute to scientific research are essential to attract, recruit and retain participants in CS projects (Asingizwe et al. 2020; Broadhurst et al. 2023; Richter et al. 2021). Volunteers do not expect monetary incentives but effort recognition (Asingizwe et al. 2020). Participation in CS projects requires a significant time commitment and endeavour to collect data and specimens when those resources are particularly rare or valuable (Guerrini and Contreras 2020). Hence, giving credit is critical (Meyer et al. 2021). Beyond simple courtesy, recognition can also enhance acknowledgement by non‐professional communities (Guerrini and Contreras 2020; Meyer et al. 2021) and also have a positive impact on data sharing (Groom et al. 2017).

Participants may be rewarded with (1) positive feedback, (2) published acknowledgements, (3) free equipment or supplies, (4) volunteer appreciation events and (5) group co‐authorship (Guerrini and Contreras 2020; Peter et al. 2021; Ward‐Fear et al. 2020). Recognition can also be given to the most active volunteers by inviting them to assist in data analysis or even to manage other community scientists (Gura 2013). Furthermore, sample tracking and explanations of how their contributions were used to produce outputs or decision‐making tools should be shared frequently, openly and transparently with participants (Haklay et al. 2021; Hall et al. 2024). A concluding workshop can be designed to provide individual feedback on their performance and acknowledge their valuable contributions (Broadhurst et al. 2023; Meyer et al. 2021; Peter et al. 2021; Suzuki‐Ohno et al. 2023).

## 9 Conclusion

DNA‐based monitoring projects should embrace the potential that CS offers, especially in the Global South, where traditional monitoring can be problematic. Combining community science with DNA‐based methods can enhance the temporal and geographic scope of biomonitoring surveys and has been shown to complement the field of taxonomy. CS involvement, along with scientific developments, could greatly enhance our understanding not only of the occurrence, population structure and genetic diversity of different taxa, but also potentially their abundance and their life stages.

DNA‐based biodiversity monitoring is rapidly evolving from person‐power collecting samples to a collaborative approach, where active participants engage throughout the entire monitoring process, fostering mutual, two‐way sharing and co‐creation of knowledge, and potentially leading to greater success in the implementation of conservation programmes. Yet, CS engagement should evolve from an effective method to collect large volumes of samples and data to a genuinely collaborative and participatory process that involves co‐design, co‐analysis and co‐discovery, and gaining access to data while prioritising the interests and needs of the communities.

## Author Contributions

All authors participated in the conception of this opinion paper. C.C. drafted the manuscript with the help of all authors. Final review and editing by C.C. and S.J.B. All authors have read the manuscript and gave final approval for publication.

## Conflicts of Interest

The authors declare no conflicts of interest.

## Supporting information


**Table S1:** Examples of DNA‐based monitoring projects that have successfully engaged CS participants in fieldwork, either by collecting individual specimens or eDNA/bulk samples.
**Table S2:** Examples of projects that could engage community scientists in monitoring using DNA‐based methods in Latin America.
**Table S3:** Examples of European projects that have benefited from the help of hobby experts.

## Data Availability

The authors have nothing to report.
