# Supplementary material for: The Role of Community Science in DNA‐Based Biodiversity Monitoring
Source: Mol Ecol. 2025 Sep 12;34(19):e70100. doi: 10.1111/mec.70100 (PMC12456123; doi:10.1111/mec.70100)
Supplement: Supplementary file 1 — Table S1: Examples of DNA‐based monitoring projects that have successfully engaged CS participants in fieldwork, either by collecting individual specimens or eDNA/bulk samples. Table S2: Examples of projects that could engage community scientists in monitoring using DNA‐based methods in Latin America. Table S3: Examples of European projects that have benefited from the help of hobby experts. [file MEC-34-e70100-s001.docx]

**Supplementary Table 1.** Examples of DNA-based monitoring projects that have successfully engaged CS participants in fieldwork, either by collecting individual specimens or eDNA/bulk samples.

| **Projects** | **References** | **Key role of CS participants** |
| --- | --- | --- |
| Biodiversity assessment in coastal marine environments | [(Agersnap et al., 2022)](https://www.zotero.org/google-docs/?k9FSFx) | 360 community scientists were engaged in collecting filtered seawater samples from 100 sites across Denmark over two seasons to map coastal fish biodiversity. |
|  | [(Burian et al., 2023)](https://www.zotero.org/google-docs/?P8taBf) | Scientific personnel and trained community scientists collected both seawater and freshwater samples from 47 sites in Mozambique to monitor fish communities. |
|  | [(Chaves et al., 2023)](https://www.zotero.org/google-docs/?e6XvrA) | The [Galapagos Genetic Code](https://www.galapagosscience.org/the-most-ambitious-citizen-science-project-developed-to-catalog-biodiversity-in-galapagos/) project was launched during the COVID-19 pandemic. It recruited and trained 79 people to catalogue the biodiversity of the Galapagos Islands, Ecuador. |
|  | [(Kondoh et al., 2024)](https://www.zotero.org/google-docs/?pbYaD4) | Introduction to different CS initiatives, such as [ANEMONE](https://db.anemone.bio/), carried out in Japan for biodiversity monitoring purposes. |
|  | [(Kvalheim et al., 2024)](https://www.zotero.org/google-docs/?C7Hr4A) | 32 participants collected 96 water samples from a Norwegian fjord, which led to the identification of undocumented species and the rediscovery of others. |
|  | [(Leerhøi et al., 2024)](https://www.zotero.org/google-docs/?vly4pf) | Description of the co-designed project *DNA & Life* that engaged high school students in collecting and analysing marine samples for biodiversity monitoring in Denmark. |
|  | [(Neave et al., 2023)](https://www.zotero.org/google-docs/?YtsTRU) | Scuba divers and snorkellers engaged in collecting eDNA samples to monitor inshore fish communities in England. |
|  | [(Pochon, 2025)](https://www.zotero.org/google-docs/?hbPr0g) | [Citizens of the Sea](https://www.citizensofthesea.org/) is an initiative that engages sailors, government agencies, NGOs, and Indigenous communities in monitoring oceanic regions in Oceania by collecting eDNA and environmental data. |
|  | [(SAC-TUN, 2022)](https://www.zotero.org/google-docs/?JsOXAe) | SAC-TUN, a private company, together with conservation and governmental organisations, launched a sustainable fishing initiative in Quintana Roo, Mexico. Fishers have been trained to collect water samples and fish tissue to assess fish diversity and abundance. |
|  | (Smith et al., 2024) | 40 volunteers, including snorkel and SCUBA divers, collected water samples, seagrass and algal specimens in the Great Barrier Reef Marine Park and the Coral Sea Marine Park, Australia, as part of the activities organised by the tourism company Coral Expeditions. |
|  | [(Suzuki‐Ohno et al., 2023)](https://www.zotero.org/google-docs/?vgx2g4) | 168 participants collected eDNA samples over two seasons to describe the marine fish biodiversity in coastal areas along Japan. |
|  | [(Suominen et al., 2024)](https://www.zotero.org/google-docs/?XJqmnX) | Description of UNESCO’s eDNA Expeditions that have involved over 250 community scientists in the inventory of marine biodiversity at 21 UNESCO’s World Heritage marine sites. |
|  | [(Takahashi et al., 2023)](https://www.zotero.org/google-docs/?nFS0p5) | Description of the Sequence our Seas (SoS) program, which engaged school and community groups in eDNA sampling of local marine environments in Australia. |
| Biodiversity assessment in urban environments | [(Horvath, 2023; Márton et al., 2025)](https://www.zotero.org/google-docs/?PvxYRZ) | [MyPond](https://mypond.hu/en/) was a CS program that engaged pond owners to collect water samples to assess the biodiversity of garden ponds in Hungary. |
|  | [(Zhang et al., 2023)](https://www.zotero.org/google-docs/?oQQf7q) | 44 high school students were engaged in sampling water and sediment from urban wetlands to monitor multitrophic biodiversity in Nanjing, China. |
| Biodiversity assessment in freshwater environments | [(Broadhurst et al., 2023)](https://www.zotero.org/google-docs/?msCM24) | The paper strongly emphasises the motivations and experiences of community scientists who collected water samples to monitor mammals in Essex, England. |
|  | [(Brown & De Brauwer, 2025)](https://www.zotero.org/google-docs/?tujJhE) | Description of a monitoring program in the estuaries of northern New South Wales, Australia, co-created and implemented by the Yaegl People. |
|  | (Bunce & Freeth, 2022) | The [Wai Tuwhera o te Taiao](https://www.epa.govt.nz/community-involvement/open-waters-aotearoa/) programme (Open Waters Aotearoa), developed by the Environmental Protection Authority New Zealand, involves community groups to collect water samples to protect, monitor and restore freshwater ecosystems across the country. |
|  | [(Clarke et al., 2023)](https://www.zotero.org/google-docs/?hpzUgQ) | Description of a co-design project that involved volunteers monitoring the Bure River in Norfolk, England, for several years to better understand how anthropogenic pressures affect fish biodiversity. |
|  | [(Couceiro et al., 2022)](https://www.zotero.org/google-docs/?OQiloY) | The GB Row Challenge engaged rowing teams in collecting eDNA samples around the coastline of Great Britain to map the British marine biodiversity. |
|  | [(Couton et al., 2023)](https://www.zotero.org/google-docs/?TPhvqD) | Municipal water providers collected water samples and captured organisms in 20 sites in the catchment basin of the river Töss to describe the distribution and abundance of groundwater amphipods in Switzerland. |
|  | (EnviroDNA, 2019) | 30 landholders and landcare locals collected water samples from 18 farm dams across West Gippsland, Australia to detect wildlife in their properties. |
|  | [(Larsen, 2024; Stammnitz et al., 2024)](https://www.zotero.org/google-docs/?MGAltM) | The [LeDNA](https://environmental-dna.ethz.ch/research/ercledna.html) and [PyriSentinel](https://pyrisentinel.eu/) projects aim to conduct eDNA biodiversity monitoring of numerous low- and high-altitude lakes in more than 80 countries and the Pyrenees, respectively, with samples collected both by researchers and community scientists. |
|  | [(Robinson et al., 2021)](https://www.zotero.org/google-docs/?Tgk926) | Description of the CS project [STREAM](https://livinglakescanada.ca/project/stream-dna-metabarcoding/) implementation, which involves communities in monitoring freshwater ecosystems in Canada. |
|  | [(Thalinger et al., 2023)](https://www.zotero.org/google-docs/?9xSoAZ) | Case study showing the importance of community-based sampling campaigns to monitor large Canadian river systems. |
|  | [(Wilken, 2018)](https://www.zotero.org/google-docs/?NpJ7iZ) | Description of a CS initiative carried out by the Natural History Museum of Denmark as part of the DNA & Life project that involved high school students in eDNA monitoring of lakes. |
|  |  | The [1000Rivers](https://1000rivers.net/) is a project launched by NatureMetrics, theUniversity of Hull and theUniversity of the Highlands and Islands, aiming to monitor waterways throughout the North Atlantic region |
|  |  | The [World Wide Fund for Nature (WWF)](https://www.wwf.org.pe/?361095/Citizen-Science-sustainable-tourism-contributes-to-the-conservation-of-river-dolphins-and-other-aquatic-species-in-Northern-Amazon) launched a project where tourist operators and tourists in the Peruvian Amazon were engaged to collect water samples to monitor vertebrate species. |
|  |  | [eBioAtlas](https://ebioatlas.org/) is a project by NatureMetrics and IUCN aimed at creating a global atlas of biodiversity in the world’s river basins, involving local stakeholders and citizen scientists. |
|  |  | The project “[DNA macht Schule](https://www.dna-macht-schule.de/)”, developed by the University of Duisburg-Essen, involves school students who collect water samples across North Rhine-Westphalia, Germany, to understand the ecological status of waterbodies. |
|  |  | The [eDNA Citizen Science](https://www.e3dna.com/citizenscience) (e^3^DNA) project started in March 2025 and aims to involve local stakeholders in the biomonitoring of aquatic and terrestrial ecosystems. Soil, water, and air samples will be collected across The Netherlands. The project is led by the University of Leiden. |
| Biodiversity assessment in soil ecosystems | [(Meyer et al., 2021)](https://www.zotero.org/google-docs/?WH85QT) | The [CALeDNA](https://ucedna.com/) project aimed at inventorying biodiversity in California across kingdoms. More than 1000 community science volunteers have been engaged in collecting soil and sediment samples using the provided sampling kits for eDNA metabarcoding. |
|  | [(So et al., 2022)](https://www.zotero.org/google-docs/?Dlhxmr) | Secondary school teachers and students were engaged in characterising the soil macrofauna in Hong Kong. |
|  | [(Crous et al., 2021)](https://www.zotero.org/google-docs/?wcv3Oh) | [Fungi for the Future](https://fungiforthefuture.org/) is a CS project launched by the Westerdijk Fungal Biodiversity Institute and the Utrecht University Museum aiming to inventorise and discover new fungi in the Netherlands. |
|  |  | [SoilRise](https://www.soilrise.eu/home/) is an ongoing project aiming to assess earthworm diversity in Europe with the help of land managers, stakeholders and volunteers. |
| Insect monitoring | (Centre of Biodiversity Genomics, 2017) | The [Global Malaise Program](https://biodiversitygenomics.net/projects/gmp/) was an international collaboration that involved the participation of different stakeholders to assess terrestrial arthropod communities worldwide. 33 out of 49 countries belong to the Global South. |
|  | (D’Souza et al., 2021) | Staff and rangers working at Kruger National Park, South Africa were responsible for the collection of Malaise traps. |
|  | (Slater-Baker et al., 2025) | The ‘[Insect Investigators](https://insectinvestigators.com.au/)’ involved students and teachers of 50 regional schools in Australia in the collection of arthropods using Malaise traps. |
|  | [(Steinke et al., 2017)](https://www.zotero.org/google-docs/?R1BlA0) | The [School Malaise Trap Program](https://malaiseprogram.com/) allowed thousands of students to explore insect diversity at their schoolyards in Canada. |
|  | [(Lehmann et al., 2021)](https://www.zotero.org/google-docs/?iiql1R) | Community scientists in the [DINA project](https://storymaps.arcgis.com/stories/4e24dc33f079481385de35b72587186e) were involved in managing Malaise traps to measure insect diversity in nature-protected areas and arable lands in Germany. |
|  | [(Svenningsen et al., 2021)](https://www.zotero.org/google-docs/?nzHHVx) | 151 volunteers collected insects with nets attached to the rooftops of cars to measure the richness and occurrence of flying insects in different land cover types of Denmark. |
|  |  | The [Fibras](https://proyectos.humboldt.org.co/fibras/) and Biomonitores programmes, led by the Humboldt Institute and Ecopetrol in Colombia, engage local communities and university students in collecting insects for DNA barcoding. Capacity building is also offered to participants. |
|  |  | [BeeCode](https://impetus4cs.eu/beecode-berlin/) is a project based in Berlin, Germany, aiming to study the genetic diversity of honeybee subspecies in the city. Beekeepers and students are involved in the collection of bee samples, which can be processed and analysed in community spaces using a mobile genetic laboratory. |
| Single-species monitoring | [(Ayre et al., 2025; Roberts et al., 2025)](https://www.zotero.org/google-docs/?GKTX6v) | Description of the [Great Australian Wildlife Search](https://www.wildlifesearch.org.au/) program to monitor aquatic environments in various river basins. Over 200 volunteers were engaged to collect eDNA samples to gather information on Australian species, such as platypuses and growling grass frogs. |
|  | [(Biggs et al., 2015)](https://www.zotero.org/google-docs/?h7jcfA) | Volunteers were recruited to collect eDNA samples to monitor great crested newts in the UK. |
|  | [(Bonicalza et al., 2024; Valsecchi et al., 2023)](https://www.zotero.org/google-docs/?Pz7miv) | Volunteers and non-profit organisations collected water samples at different sites of the Mediterranean Sea to monitor Mediterranean monk seals. |
|  | (Coleman et al., 2024) | First Nation environmental rangers and Traditional Owners engaged in eDNA water sampling to monitor short-finned eel. |
|  | [(Corro et al., 2023)](https://www.zotero.org/google-docs/?0nV7Vq) | Training and development of eDNA and sequencing methods for citizen-mycologists offered by the [Wild Fungi DNA project](https://myco.org.au/wild-dna-project/) with the key aim of increasing the taxonomic knowledge of Australian fungi. |
|  | [(Knudsen et al., 2023)](https://www.zotero.org/google-docs/?Mfnalv) | High school students collected and analysed water samples to detect eDNA from 14 amphibian species in Denmark. |
|  |  | The [PUKI](https://www.puki.hhu.de/) project, based in Düsseldorf, Germany, monitors five species of crucifer plants to understand their genetic adaptations to climate change. Volunteers engage in the collection of leaves and soil samples, along with taking measurements of the plants. Moreover, biology teachers have the opportunity to learn about molecular methods to integrate the project into school curricula. |
|  |  | [Bat Conservation International](https://www.batcon.org/citizen-science-edna-project-faqs/) is currently engaging volunteers in Arizona, USA, to collect water samples from hummingbird feeders to monitor Mexican long-nosed bats. |
| Population genetic diversity | [(Cunningham‐Eurich et al., 2023)](https://www.zotero.org/google-docs/?qDMffR) | Participants collected social wasps as part of the Big Wasp Survey, which aimed to map the diversity and distribution of vespine wasps in the UK. |
|  | [(von Thaden et al., 2022)](https://www.zotero.org/google-docs/?tYUedh) | Community scientists collected samples of garden dormice across Europe to infer regional phylogeography and define distinct conservation units using RADseq. |
| Detection of invasive alien species | [(Brown et al., 2018)](https://www.zotero.org/google-docs/?NYE3WL) | Description of the [Harlequin Ladybird Survey](https://coleoptera.org.uk/coccinellidae/home) that included community observations of the Harlequin ladybird in the UK and Ireland from 2003 to 2016. |
|  | [(Sheard et al., 2020)](https://www.zotero.org/google-docs/?EmiIX7) | Description of the Ant Hunt CS project that engaged children and accompanied grown-ups in collecting ants on bait cards to determine the presence of an exotic ant in Denmark. |
|  | [(Zirngibl et al., 2022)](https://www.zotero.org/google-docs/?bRL8gS) | The authors developed and validated an easy-to-use Recombinase Polymerase Amplification Lateral Flow assay that can be applied by volunteers to detect Mediterranean fanworms in New Zealand. |
| Detection of hybridisation | [(Tyagi et al., 2023)](https://www.zotero.org/google-docs/?d9qvi5) | A group of nature enthusiasts collected non-invasive samples of two suspected wolf-dog hybrids that were confirmed with ddRAD sequencing. |
| Pathogen monitoring | (Shan et al., 2023) | CS participants collected ticks using special kits across Great Britain to detect *Borrelia* species by PCR methods. |

**Supplementary Table 2.** Examples of projects that could engage community scientists in monitoring using DNA-based methods in Latin America.

| **Project** | **Country** | **Supported by** | **Description** |
| --- | --- | --- | --- |
| [Surveying Biodiversity for Amazon Rainforest Conservation](https://swissnex.org/brazil/news/eth-biodivx-surveying-biodiversity-for-amazon-rainforest-conservation/)  (GainForest, 2024; Geckeler et al., 2025) | Brazil | Switzerland | This project was part of a XPRIZE rainforest competition, where researchers collected water samples and eDNA from the canopy using drones to survey the biodiversity of the Amazon rainforest. They trained indigenous communities to use drones as well as developed sampling eDNA backpack labs for eventual CS purposes. |
| Amazon Water Alliance | Bolivia, Ecuador, Peru, Brazil, Colombia | USA, France | Different stakeholders and local communities have partnered to identify and complete the inventory of the ichthyofauna in the Amazon basin using eDNA. So far, community scientists are involved in recording species observations. |
| BioAlfa  (Janzen & Hallwachs, 2019) | Costa Rica | USA | This project will aim to DNA barcode all biodiversity in the country and continue supporting the labour of parataxonomists. |

**Supplementary Table 3**. Examples of European projects that have benefited from the help of hobby experts.

| **Projects** | **Description** |
| --- | --- |
| [GBOL](https://gbol.bolgermany.de/en/german-barcode-of-life-2/) (German Barcode of Life) | These projects depended on a network of privately acting taxonomic experts, which contributed to fresh sampling, preservation, and identification of organisms for DNA barcoding. More than 300 external hobby experts voluntarily registered and obtained sampling kits and the relevant metadata sheets through the GBOL web portal, and benefited in turn from a financial incentive, free access to sequence data and metadata, and the possibility of becoming co-authors in joint publications [(Geiger et al., 2016)](https://www.zotero.org/google-docs/?Gcf3Kf). |
| [ARISE](https://www.arise-biodiversity.nl/) (Authoritative and Rapid Identification System for Essential Biodiversity Information) | This project currently engages hobby experts in collecting and identifying Dutch species to create a reference database that will contain validated DNA barcodes for every multicellular species in the Netherlands, together with photos and physical vouchers [(Van Ommen Kloeke et al., 2022)](https://www.zotero.org/google-docs/?4Y6UUC). Sampling kits, videos, digital apps and platforms, and engagement events were created to encourage hobby experts while improving the standardisation of methods and data capture. To date, this has already led to the contribution of around 20,000 identified specimens and will increase further while workflows are optimised for speed, cost and user-friendliness. Hobby experts, in return, get referenced for their contributions, free participation in engagement events and access to the sequence data. For those trying to understand their favourite species, this gives a genuine opportunity for understanding genetic differences between species and, in some cases, even discovering new species for science or their country. |
| [BGE](https://biodiversitygenomics.eu/) (Biodiversity Genomics Europe) | This Horizon-funded project has involved taxon experts in species inventories, especially of pollinators, freshwater and marine species, and in the curation of European reference databases that can be used for DNA metabarcoding and biomonitoring purposes. More specifically, community scientists have been involved in collecting insect community samples through the operation of Malaise traps and collection of aquatic eDNA from ports and harbours to detect marine non-indigenous species. In both cases, detailed collection protocols and sampling kits were supplied by the project to ensure as robust sampling as possible. Community scientists were further trained through face-to-face meetings or through an instructive video if in-person gatherings were impractical to arrange. |
| [Barcoding the Broads](https://www.earlham.ac.uk/barcoding-broads) | It is a Wellcome-funded initiative of the Darwin Tree of Life project led by the Earlham Institute. It engages naturalists in identifying rare species found in the Norfolk Broads, UK, while providing DNA barcoding training. |
| [SPRING](https://www.ufz.de/spring-pollination/index.php?en=49053) (Strengthening Pollinator Recovery through INdicators and monitorinG) | It focused on pollinator monitoring in Europe and one of its aims was to provide taxonomic training and tools through the [PollinatorAcademy](https://pollinatoracademy.eu/) to increase the number of experts in pollinator species. |
| [EDIT](https://cordis.europa.eu/project/id/18340) (European Distributed Institute of Taxonomy) | It was a CETAF (Consortium of European Taxonomic Facilities) conservation initiative that resulted in the creation of the [Distributed European School of Taxonomy](https://cetaf.org/explore/dest-distributed-school-of-european-taxonomy/), which offers training opportunities to anyone interested in a taxonomic career. |
| [TETTRIs](https://tettris.eu/) (Transforming European Taxonomy through Training, Research, and Innovations) | It is a Horizon-funded project that started in 2022. Its goal is to develop training programmes to enhance taxonomic expertise among professional taxonomists, hobby experts and community scientists [(Willemse & Laakkonnen, 2024)](https://www.zotero.org/google-docs/?IRE2ZA). Additionally, TETTRIs has established the Taxonomy Recognition Day on May 23rd to highlight the importance of taxonomy in biodiversity among stakeholders and the general public [(TETTRIs, 2024)](https://www.zotero.org/google-docs/?cHidDc) |

**References**

[Agersnap, S., Sigsgaard, E. E., Jensen, M. R., Avila, M. D. P., Carl, H., Møller, P. R., Krøs, S. L., Knudsen, S. W., Wisz, M. S., & Thomsen, P. F. (2022). A National Scale “BioBlitz” Using Citizen Science and eDNA Metabarcoding for Monitoring Coastal Marine Fish. *Frontiers in Marine Science*, *9*, 824100. https://doi.org/10.3389/fmars.2022.824100](https://www.zotero.org/google-docs/?gRONjd)

[Ayre, B., McNeil, D., Kaminskas, S., Weeks, A., Marwood, S., Thurtell, L., Wood, B., & Carroll, N. (2025). *Using citizen science to monitor aquatic biodiversity across a million square kilometres*. 2nd Australian and New Zealand eDNA Conference, Wellington, New Zealand. https://ednaconference.com.au/5712](https://www.zotero.org/google-docs/?gRONjd)

[Biggs, J., Ewald, N., Valentini, A., Gaboriaud, C., Dejean, T., Griffiths, R. A., Foster, J., Wilkinson, J. W., Arnell, A., Brotherton, P., Williams, P., & Dunn, F. (2015). Using eDNA to develop a national citizen science-based monitoring programme for the great crested newt (Triturus cristatus). *Special Issue: Environmental DNA: A Powerful New Tool for Biological Conservation*, *183*, 19–28. https://doi.org/10.1016/j.biocon.2014.11.029](https://www.zotero.org/google-docs/?gRONjd)

[Bonicalza, S., Valsecchi, E., Coppola, E., Catapano, V., & Thatcher, H. (2024). Citizen science in eDNA monitoring for Mediterranean monk seal conservation. *BMC Ecology and Evolution*, *24*(1), 148. https://doi.org/10.1186/s12862-024-02338-8](https://www.zotero.org/google-docs/?gRONjd)

[Broadhurst, H., Smith, E. E., Jackman, J. M., Singleton, N., Tansley, D., Raynor, R., Sales, N. G., Ochu, E., & McDevitt, A. D. (2023). *Citizen scientists’ motivation to participate in environmental DNA (eDNA) surveys: A case study on monitoring mammals in the UK*. https://doi.org/10.31235/osf.io/fa83k](https://www.zotero.org/google-docs/?gRONjd)

[Brown, G., & De Brauwer, M. (2025). *Estuarine monitoring and restoration in Yaegl country (NSW, Australia)*. 2nd Australian and New Zealand eDNA Conference, Wellington, New Zealand. https://ednaconference.com.au/5583](https://www.zotero.org/google-docs/?gRONjd)

[Brown, P. M. J., Roy, D. B., Harrower, C., H. J. Dean, Rorke, S. L., & Roy, H. E. (2018). Spread of a model invasive alien species, the harlequin ladybird Harmonia axyridis in Britain and Ireland. *Scientific Data*, *5*(1), 180239. https://doi.org/10.1038/sdata.2018.239](https://www.zotero.org/google-docs/?gRONjd)

Bunce, M., & Freeth, A. (2022). Looking further and deeper into environmental protection, regulation and policy using environmental DNA (eDNA). *Policy Quarterly*, *18*, 33–39. https://doi.org/10.26686/pq.v18i4.8013

[Burian, A., Bruce, K., Tovela, E., Bakker, J., Balcells, L., Bennett, R., Chordekar, S., Costa, H. M., Crampton‐Platt, A., de Boer, H., Ross‐Gillespie, V., de Sacramento, A., Sidat, N., Simbine, L., Ready, J., Tang, C., & Mauvisseau, Q. (2023). Merging two eDNA metabarcoding approaches and citizen‐science‐based sampling to facilitate fish community monitoring along vast Sub‐Saharan coastlines. *Molecular Ecology Resources*, *23*(7), 1641–1655. https://doi.org/10.1111/1755-0998.13839](https://www.zotero.org/google-docs/?gRONjd)

Centre of Biodiversity Genomics. (2017). *Global Malaise Program. Progress Report*. https://biodiversitygenomics.net/wp-content/uploads/2023/10/GMP-Progress-Report-2017.pdf

[Chaves, J. A., Bonneaud, C., Russell, A., Mena, C. F., Proaño, C., Ortiz, D. A., Cruz, M., Velez, A., Jones, J. S., Chaigneau, T., & Pazmino, D. A. (2023). Galapagos Genetic Barcode: A Model for Island Economic Resilience During the COVID-19 Pandemic. In S. J. Walsh, C. F. Mena, J. R. Stewart, & J. P. Muñoz Pérez (Eds.), *Island Ecosystems: Challenges to Sustainability* (pp. 453–468). Springer International Publishing. https://doi.org/10.1007/978-3-031-28089-4_29](https://www.zotero.org/google-docs/?gRONjd)

[Clarke, S. J., Long, E., Biggs, J., Bruce, K., Weatherby, A., Harper, L. R., & Hails, R. S. (2023). Co‐design of a citizen science study: Unlocking the potential of eDNA for volunteer freshwater monitoring. *Ecological Solutions and Evidence*, *4*(3), e12273. https://doi.org/10.1002/2688-8319.12273](https://www.zotero.org/google-docs/?gRONjd)

Coleman, H. T., Sherriff, A., & Hudson, N. (2024). Blending Indigenous science with molecular techniques using Environmental DNA. *Proceedings of the 11th Australian Stream Management Conference*.

[Corro, E., McIntyre, E., Xu, M., & McMullan-Fisher, S. (2023). *Using citizen science and new DNA technology to rapidly increase our baseline knowledge of fungal ecology*. First Australian and New Zealand eDNA Conference, Tasmania, Australia. https://ednaconference.com.au/4302](https://www.zotero.org/google-docs/?gRONjd)

[Couceiro, F., Trayford, J., Lundgren, A., Ford, A., Bruce, K., Carey, A., & Mowat, R. (2022). *GB Row 2022—Impact Report*. https://www.port.ac.uk/sites/default/files/2023-03/GB%20Row%202022%20impact%20report.pdf](https://www.zotero.org/google-docs/?gRONjd)

[Couton, M., Studer, A., Hürlemann, S., Locher, N., Knüsel, M., Alther, R., & Altermatt, F. (2023). Integrating citizen science and environmental DNA metabarcoding to study biodiversity of groundwater amphipods in Switzerland. *Scientific Reports*, *13*(1), 18097. https://doi.org/10.1038/s41598-023-44908-8](https://www.zotero.org/google-docs/?gRONjd)

[Crous, P. W., Hernández-Restrepo, M., Van Iperen, A. L., Starink-Willemse, M., Sandoval-Denis, M., & Groenewald, J. Z. (2021). Citizen science project reveals novel fusarioid fungi (Nectriaceae, Sordariomycetes) from urban soils. *Fungal Systematics and Evolution*. https://doi.org/10.3114/fuse.2021.08.09](https://www.zotero.org/google-docs/?gRONjd)

[Cunningham‐Eurich, I., Kontou, D., Yordanova, M., Maeda‐Obregon, A., Favreau, E., Wang, J., Hart, A. G., & Sumner, S. (2023). Using citizen science data to assess the population genetic structure of the common yellowjacket wasp, *Vespula vulgaris*. *Insect Molecular Biology*, *32*(6), 634–647. https://doi.org/10.1111/imb.12862](https://www.zotero.org/google-docs/?gRONjd)

D’Souza, M. L., van der Bank, M., Shongwe, Z., Rattray, R. D., Stewart, R., van Rooyen, J., Govender, D., & Hebert, P. D. N. (2021). Biodiversity baselines: Tracking insects in Kruger National Park with DNA barcodes. *Biological Conservation*, *256*, 109034. https://doi.org/10.1016/j.biocon.2021.109034

GainForest. (2024). *2nd Annual Impact Report*. <https://www.canva.com/design/DAGNpwdK0jo/QkBOQ1gfl0gy8jDTBAo10g/view?utm_content=DAGNpwdK0jo&utm_campaign=designshare&utm_medium=link&utm_source=editor#26>

Geckeler, C., Kirchgeorg, S., Strunck, G., Thostrup, F. B., Sangermano, F., Desiderato, A., ... & Mintchev, S. (2025). Field Deployment of BiodivX Drones in the Amazon Rainforest for Biodiversity Monitoring. *IEEE Transactions on Field Robotics*.

Geiger, M. F., Astrin, J. J., Borsch, T., Burkhardt, U., Grobe, P., Hand, R., Hausmann, A., Hohberg, K., Krogmann, L., Lutz, M., Monje, C., Misof, B., Morinière, J., Müller, K., Pietsch, S., Quandt, D., Rulik, B., Scholler, M., Traunspurger, W., … Wägele, W. (2016). How to tackle the molecular species inventory for an industrialized nation—Lessons from the first phase of the German Barcode of Life initiative GBOL (2012–2015). *Genome*, *59*(9), 661–670. https://doi.org/10.1139/gen-2015-0185

[Horvath, Z. (2023). *MyPond: A citizen science project to explore the hidden biodiversity of garden ponds*. ASLO Aquatic Sciences Meeting, Palma de Mallorca, Spain.](https://www.zotero.org/google-docs/?gRONjd)

Janzen, D., & Hallwachs, W. (2019). How a tropical country can DNA barcode itself. *iBOL Barcode Bulletin*. https://doi.org/10.21083/ibol.v9i1.5526

[Knudsen, S. W., Hesselsøe, M., Rytter, M., Lillemark, M. R., Tøttrup, A. P., Rahbek, C., Sheard, J. K., Thomsen, P. F., Agersnap, S., Mortensen, P. B., & Møller, P. R. (2023). Detection of environmental DNA from amphibians in Northern Europe applied in citizen science. *Environmental DNA*, *5*(6), 1429–1448. https://doi.org/10.1002/edn3.462](https://www.zotero.org/google-docs/?gRONjd)

[Kondoh, M., Kasada, M., Abe, T., Kasai, A., Dazai, A., Masuda, R., Seino, S., Suzuki, S., Suzuki-Ohno, Y., & Tanabe, A. S. (2024). Community Science Initiatives Utilizing Environmental DNA. In Y. Suzuki-Ohno (Ed.), *Community Science in Ecology: Case Studies of Public Participation in Ecological Research in Japan* (pp. 83–99). Springer Nature Singapore. https://doi.org/10.1007/978-981-97-0304-3_6](https://www.zotero.org/google-docs/?gRONjd)

[Kvalheim, L., Stensrud, E., Knutsen, H., Hyvärinen, O., & Eiler, A. (2024). Integration of citizen science and eDNA reveals novel ecological insights for marine fish conservation. *Environmental DNA*, *6*(4), e584. https://doi.org/10.1002/edn3.584](https://www.zotero.org/google-docs/?gRONjd)

[Larsen, L. (2024). Why citizen scientists are gathering DNA from hundreds of lakes-on the same day. *Nature*.](https://www.zotero.org/google-docs/?gRONjd)

[Leerhøi, F., Rytter, M., Lillemark, M. R., Randeris, B., Rix, C., Olesen, J., Olsen, M. T., Møller, P. R., Lundholm, N., Knudsen, S. W., & Tøttrup, A. P. (2024). Exploring the potential of extreme citizen science with Danish high school students using environmental DNA for marine monitoring. *Frontiers in Marine Science*, *11*, 1347298. https://doi.org/10.3389/fmars.2024.1347298](https://www.zotero.org/google-docs/?gRONjd)

[Lehmann, G. U. C., Bakanov, N., Behnisch, M., Bourlat, S. J., Brühl, C. A., Eichler, L., Fickel, T., Geiger, M. F., Gemeinholzer, B., Hörren, T., Köthe, S., Lux, A., Meinel, G., Mühlethaler, R., Poglitsch, H., Schäffler, L., Schlechtriemen, U., Schneider, F. D., Schulte, R., … Zizka, V. M. A. (2021). Diversity of Insects in Nature protected Areas (DINA): An interdisciplinary German research project. *Biodiversity and Conservation*, *30*(8), 2605–2614. https://doi.org/10.1007/s10531-021-02209-4](https://www.zotero.org/google-docs/?gRONjd)

[Márton, Z., Barta, B., Vad, C. F., Szabó, B., HAMER, A. J., Kardos, V., Laskai, C., Fierpasz, Á., & Horvath, Z. (2025). Effects of urbanisation, habitat characteristics, and management on garden pond biodiversity: Findings from a large-scale citizen science survey. *Landscape and Urban Planning*, *257*, 105299. https://doi.org/10.1016/j.landurbplan.2025.105299](https://www.zotero.org/google-docs/?gRONjd)

[Meyer, R., Ramos, M. M., Lin, M., Schweizer, T., Gold, Z., Ramos, D. R., Shirazi, S., Kandlikar, G., Kwan, W.-Y., Curd, E., Freise, A., Parker, J. M., Sexton, J., Wetzer, R., Pentcheff, D., Wall, A., Pipes, L., Garcia-Vedrenne, A., Mejia, M. P., … Wayne, R. (2021). The CALeDNA program: Citizen scientists and researchers inventory California’s biodiversity. *California Agriculture*, *75*(1), 20–32. https://doi.org/10.3733/ca.2021a0001](https://www.zotero.org/google-docs/?gRONjd)

[Neave, E. F., Mariani, S., & Meek, S. (2023). *Passive eDNA capture by SCUBA divers and snorkellers for monitoring inshore fish biodiversity*. Natural England. https://publications.naturalengland.org.uk/publication/6249569948794880](https://www.zotero.org/google-docs/?gRONjd)

[Pochon, X. (2025). *Citizens of the Sea: Mapping ocean health at scale*. 2nd Australian and New Zealand eDNA Conference, Wellington, New Zealand. https://ednaconference.com.au/5947](https://www.zotero.org/google-docs/?gRONjd)

[Roberts, H., Weeks, A., Marwood, S., Song, S., Noble, L., Impey, R., McNeil, D., & Duncan, C. (2025). *The Great Australian Wildlife Search: Large-scale biodiversity assessments using eDNA metabarcoding and citizen scientists.* 2nd Australian and New Zealand eDNA Conference, Wellington, New Zealand. https://ednaconference.com.au/5694](https://www.zotero.org/google-docs/?gRONjd)

[Robinson, C. V., Baird, D. J., Wright, M. T. G., Porter, T. M., Hartwig, K., Hendriks, E., Maclean, L., Mallinson, R., Monk, W. A., Paquette, C., & Hajibabaei, M. (2021). Combining DNA and people power for healthy rivers: Implementing the STREAM community-based approach for global freshwater monitoring. *Perspectives in Ecology and Conservation*, *19*(3), 279–285. https://doi.org/10.1016/j.pecon.2021.03.001](https://www.zotero.org/google-docs/?gRONjd)

[SAC-TUN. (2022). *Apéndice: Estrategia Ambiental de SAC-TUN*.](https://www.zotero.org/google-docs/?gRONjd) <https://sactun.com/wp-content/uploads/2024/02/APENDICE-SACTUN-2022.pdf>

[Shan, J., Jia, Y., Hickenbotham, P., Teulières, L., & Clokie, M. R. J. (2023). Combining citizen science and molecular diagnostic methods to investigate the prevalence of Borrelia burgdorferi s.l. And Borrelia miyamotoi in tick pools across Great Britain. *Frontiers in Microbiology*, *Volume 14-2023*. https://www.frontiersin.org/journals/microbiology/articles/10.3389/fmicb.2023.1126498](https://www.zotero.org/google-docs/?iezp9g)

[Sheard, J. K., Sanders, N. J., Gundlach, C., Sämi Schär, & Rasmus Stenbak Larsen. (2020). Monitoring the influx of new species through citizen science: The first introduced ant in Denmark. *PeerJ*, *8*, e8850. https://doi.org/10.7717/peerj.8850](https://www.zotero.org/google-docs/?gRONjd)

Slater-Baker, M.-R., Fagan-Jeffries, E. P., Oestmann, K. J., Portmann, O. G., Bament, T. M., Howe, A. G., Guzik, M. T., Bradford, T. M., McClelland, A. R., Woodward, A., Clarke, S., Ducker, N., & Fernández-Triana, J. (2025). DNA barcoding, integrative taxonomy, citizen science, and Bush Blitz surveys combine to reveal 34 new species of Apanteles (Hymenoptera, Braconidae, Microgastrinae) in Australia. *ZooKeys*, *1227*, 1–128. https://doi.org/10.3897/zookeys.1227.130467

[Smith, A. K., DiBattista, J. D., Tol, S. J., Kustra, L., Stacey, J., Massey, T., & Hardisty, P. E. (2024). Assessment of Multiple Citizen Science Methods and Carbon Footprint of Tourists in Two Australian Marine Parks. *Sustainability*, *16*(24). https://doi.org/10.3390/su162411019](https://www.zotero.org/google-docs/?3QovUO)

[So, W. L., Ting, K. W., Lai, S. Y., Huang, E. Y. Y., Ma, Y., Chong, T. K., Yip, H. Y., Lee, H. T., Cheung, B. C. T., Chan, M. K., Consortium, H. K. S. B., Nong, W., Law, M. M. S., Lai, D. Y. F., & Hui, J. H. L. (2022). Revealing the millipede and other soil-macrofaunal biodiversity in Hong Kong using a citizen science approach. *Biodiversity Data Journal*, *10*, e82518. https://doi.org/10.3897/BDJ.10.e82518](https://www.zotero.org/google-docs/?gRONjd)

[Stammnitz, M. R., Hartman Scholz, A., & Duffy, D. J. (2024). Environmental DNA without borders: Let’s embrace decentralised genomics to meet the UN’s biodiversity targets. *EMBO Reports*, *25*(10), 4095–4099. https://doi.org/10.1038/s44319-024-00264-w](https://www.zotero.org/google-docs/?gRONjd)

[Steinke, D., Breton, V., Berzitis, E., & Hebert, P. D. N. (2017). The School Malaise Trap Program: Coupling educational outreach with scientific discovery. *PLOS Biology*, *15*(4), e2001829. https://doi.org/10.1371/journal.pbio.2001829](https://www.zotero.org/google-docs/?gRONjd)

[Suominen, S., Provoost, P., Principe, S., Boulanger, E., Burrows, M. T., Campoy, A. N., Costello, M. J., Earl, C., Gante, H. F., Gillard, E., Hablützel, P. I., Douvere, F., & Appeltans, W. (2024). *Engaging Communities to Safeguard Ocean Life: UNESCO Environmental DNA Expeditions*. UNESCO. https://doi.org/10.58337/CBXU3518](https://www.zotero.org/google-docs/?gRONjd)

[Suzuki‐Ohno, Y., Tanabe, A. S., Kasai, A., Masuda, R., Seino, S., Dazai, A., Suzuki, S., Abe, T., & Kondoh, M. (2023). Evaluation of community science monitoring with environmental DNA for marine fish species: “Fish survey project using environmental DNA ”. *Environmental DNA*, *5*(3), 613–623. https://doi.org/10.1002/edn3.425](https://www.zotero.org/google-docs/?gRONjd)

[Svenningsen, C. S., Frøslev, T. G., Bladt, J., Pedersen, L. B., Larsen, J. C., Ejrnæs, R., Fløjgaard, C., Hansen, A. J., Heilmann-Clausen, J., Dunn, R. R., & Tøttrup, A. P. (2021). Detecting flying insects using car nets and DNA metabarcoding. *Biology Letters*, *17*(3), 20200833. https://doi.org/10.1098/rsbl.2020.0833](https://www.zotero.org/google-docs/?gRONjd)

[Takahashi, M., Herbert, S., & Bunce, M. (2023). *Sequence our Seas (SoS): A community program to introduce eDNA at high schools in Western Australia*. First Australian and New Zealand eDNA Conference, Tasmania, Australia. https://ednaconference.com.au/4642](https://www.zotero.org/google-docs/?gRONjd)

TETTRIs. (2024). *Report Taxonomy Recognition Day*. https://tettris.eu/wp-content/uploads/2024/08/Report-of-Taxonomy-Recognition-Day.docx.pdf

[Thalinger, B., Holman, L., MacDonald, S., Cowperthwaite, M., & Steinke, D. (2023). *Studying fish biodiversity in Canadian rivers by engaging local communities in environmental DNA monitoring*. First Australian and New Zealand eDNA Conference, Tasmania, Australia. https://ednaconference.com.au/4440](https://www.zotero.org/google-docs/?gRONjd)

[Tyagi, A., Godbole, M., Vanak, A. T., & Ramakrishnan, U. (2023). Citizen science facilitates first ever genetic detection of wolf‐dog hybridization in Indian savannahs. *Ecology and Evolution*, *13*(5), e10100. https://doi.org/10.1002/ece3.10100](https://www.zotero.org/google-docs/?gRONjd)

[Uche-Dike, R., Tolman, E., Benischek, C., Schneider, M., Kohli, M. K., Bush, J., Frandsen, P. B., Errigo, I. M., Frankel, W., Gnojewski, K., Chmura, K., Jordan, D., Kittler, H., Liao, M., Tobin, T., Su, C., Castillo, G., Derderian, E., Wei, M., … Beatty, C. D. (2024). Environmental DNA vs. Community Science: Strengths and Limitations for Urban Odonata Surveys. *bioRxiv*, 2024.11.26.625270. https://doi.org/10.1101/2024.11.26.625270](https://www.zotero.org/google-docs/?gRONjd)

[Valsecchi, E., Tavecchia, G., Boldrocchi, G., Coppola, E., Ramella, D., Conte, L., Blasi, M., Bruno, A., & Galli, P. (2023). Playing “hide and seek” with the Mediterranean monk seal: A citizen science dataset reveals its distribution from molecular traces (eDNA). *Scientific Reports*, *13*(1), 2610. https://doi.org/10.1038/s41598-023-27835-6](https://www.zotero.org/google-docs/?gRONjd)

Van Ommen Kloeke, E., Huijbers, C., Beentjes, K., Kamminga, J., Bakker, P., & Kissling, W. (2022). ARISE: Building an infrastructure for species recognition and biodiversity monitoring in the Netherlands. *Biodiversity Information Science and Standards*, *6*, e93613. https://doi.org/10.3897/biss.6.93613

[von Thaden, A., Sven Büchner, Lang, J., Meinig, H., & Nowak, C. (2022). Combining citizen science and conservation genomics to reveal the causes of rapid population decline in the Garden Dormouse (Eliomys quercinus). *ARPHA Conference Abstracts*, *5*, e84437. https://doi.org/10.3897/aca.5.e84437](https://www.zotero.org/google-docs/?gRONjd)

[Wilken, U. (2018). Lakes, Labs and Learning: How an Environmental DNA Citizen Science Project Makes Sense for High School Students, Researchers and Environmental Managers. *K-12 STEM Education*, *4*(4), 391–399.](https://www.zotero.org/google-docs/?gRONjd)

[Willemse, L., & Laakkonnen, H. (2024). *Policy Brief 1: The power of reference collections in biodiversity monitoring. Deliverable 8.4. TETTRIs project.* https://tettris.eu/wp-content/uploads/2024/08/TETTRIs-D8.4-Policy-Brief-1_-The-power-of-reference-collections-in-biodiversity-monitoring.pdf](https://www.zotero.org/google-docs/?x1KJsR)

[Zhang, H., Yang, J., Zhang, L., Gu, X., & Zhang, X. (2023). Citizen science meets eDNA: A new boom in research exploring urban wetland biodiversity. *Environmental Science and Ecotechnology*, *16*, 100275. https://doi.org/10.1016/j.ese.2023.100275](https://www.zotero.org/google-docs/?gRONjd)

[Zirngibl, M., von Ammon, U., Pochon, X., & Zaiko, A. (2022). A Rapid Molecular Assay for Detecting the Mediterranean Fanworm Sabella spallanzanii Trialed by Non-Scientist Users. *Frontiers in Marine Science*, *9*. https://www.frontiersin.org/journals/marine-science/articles/10.3389/fmars.2022.861657](https://www.zotero.org/google-docs/?gRONjd)
